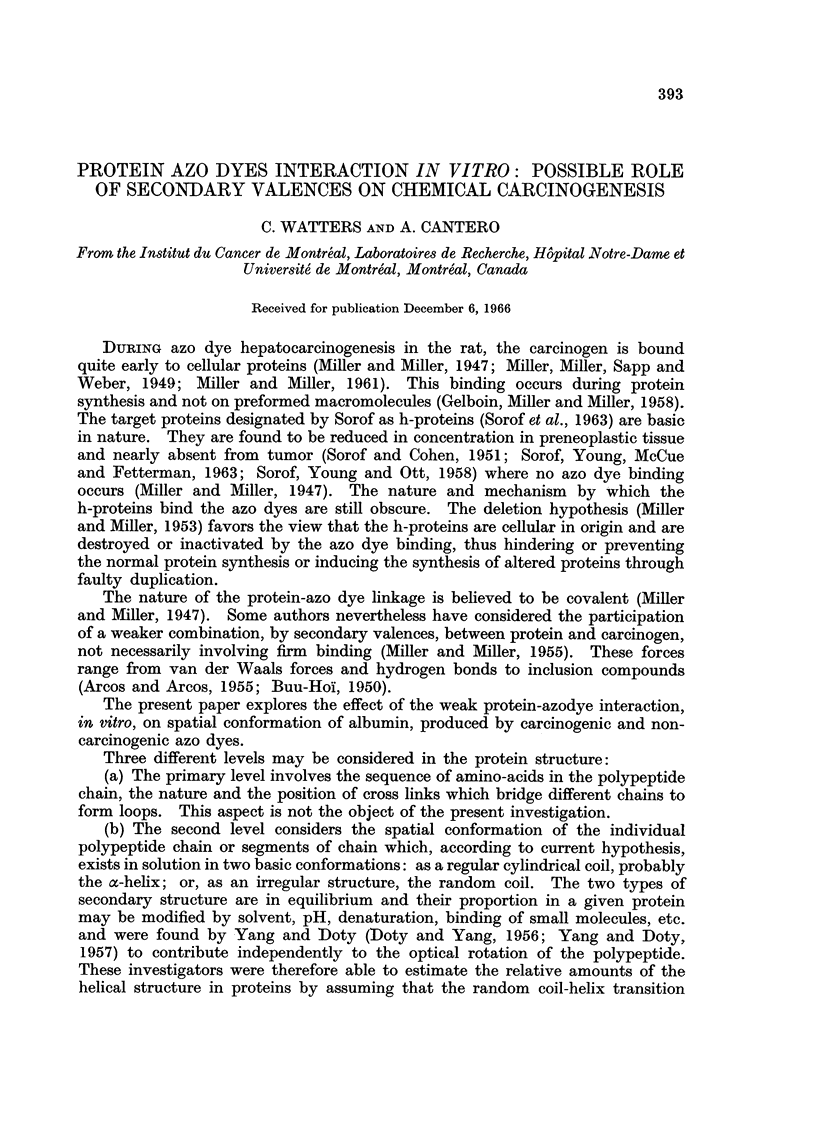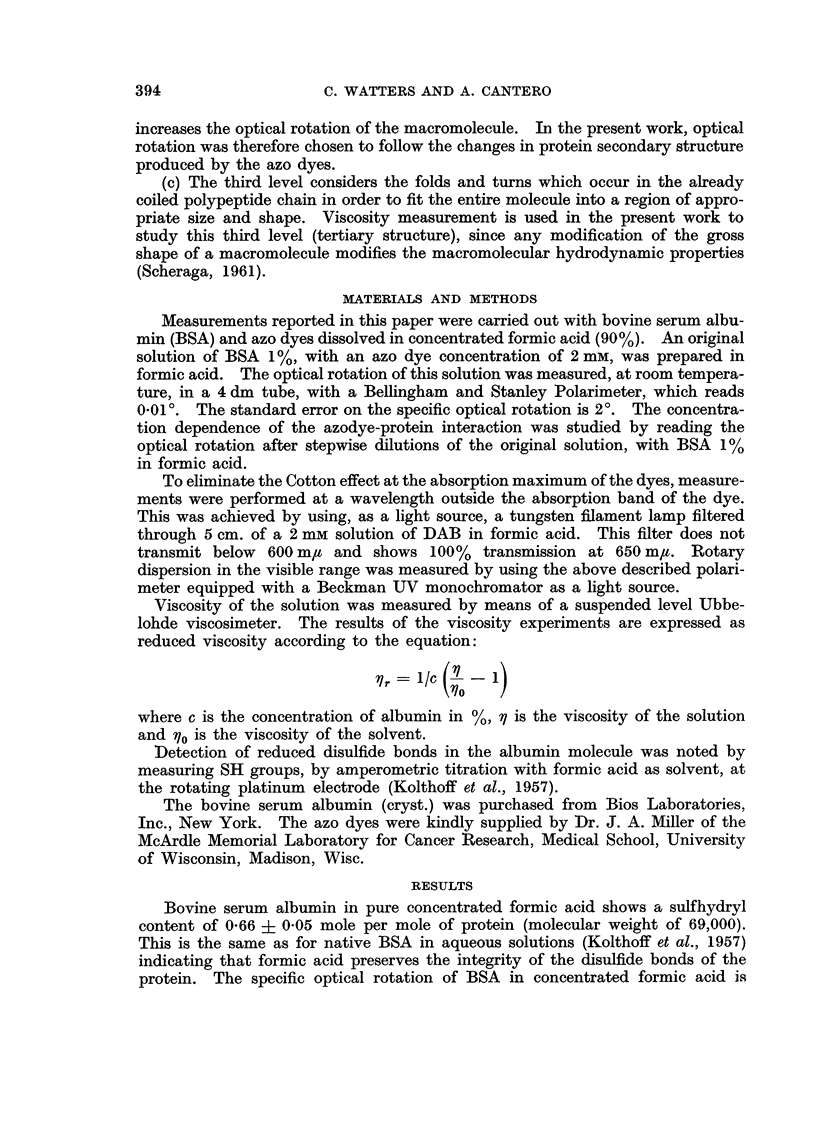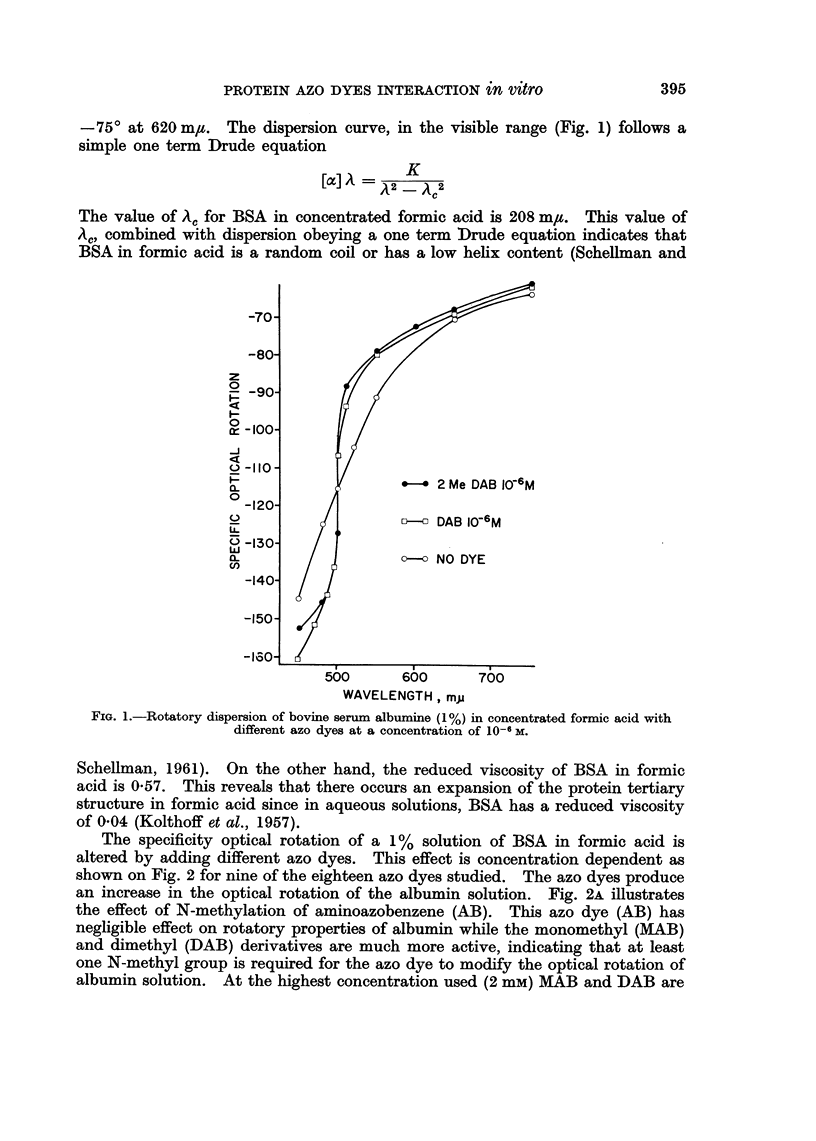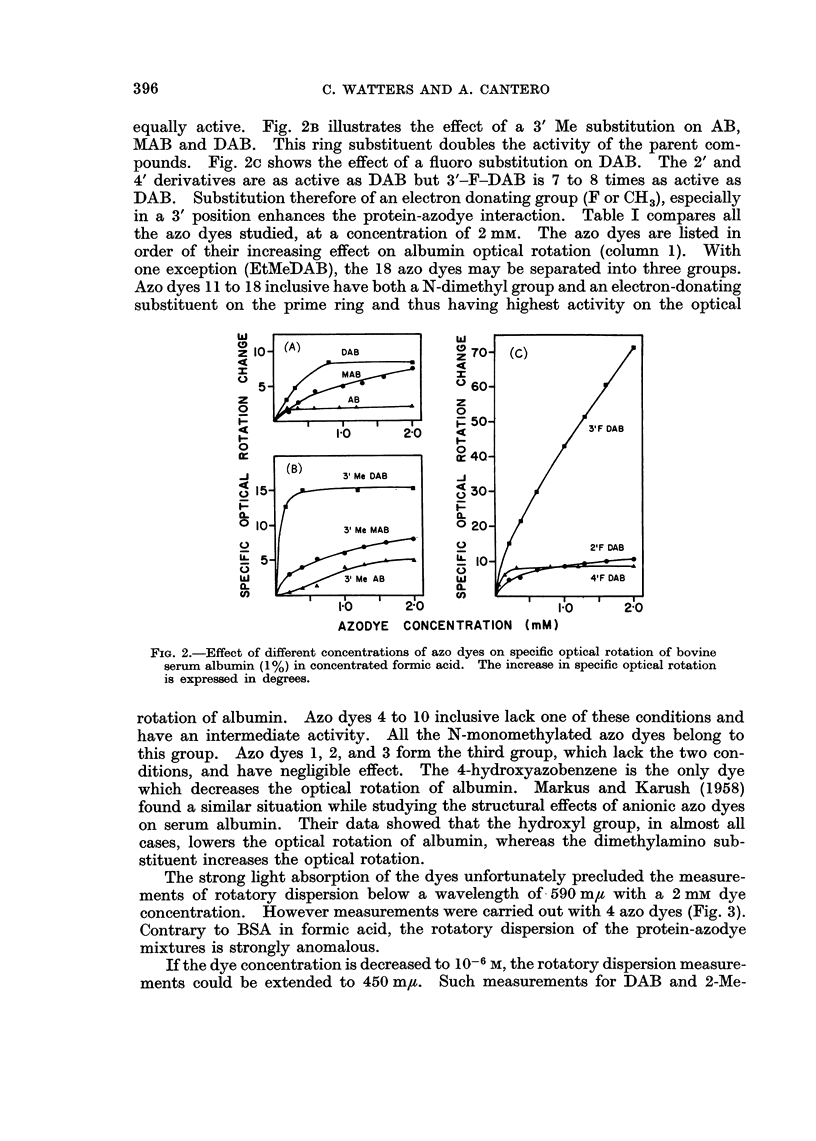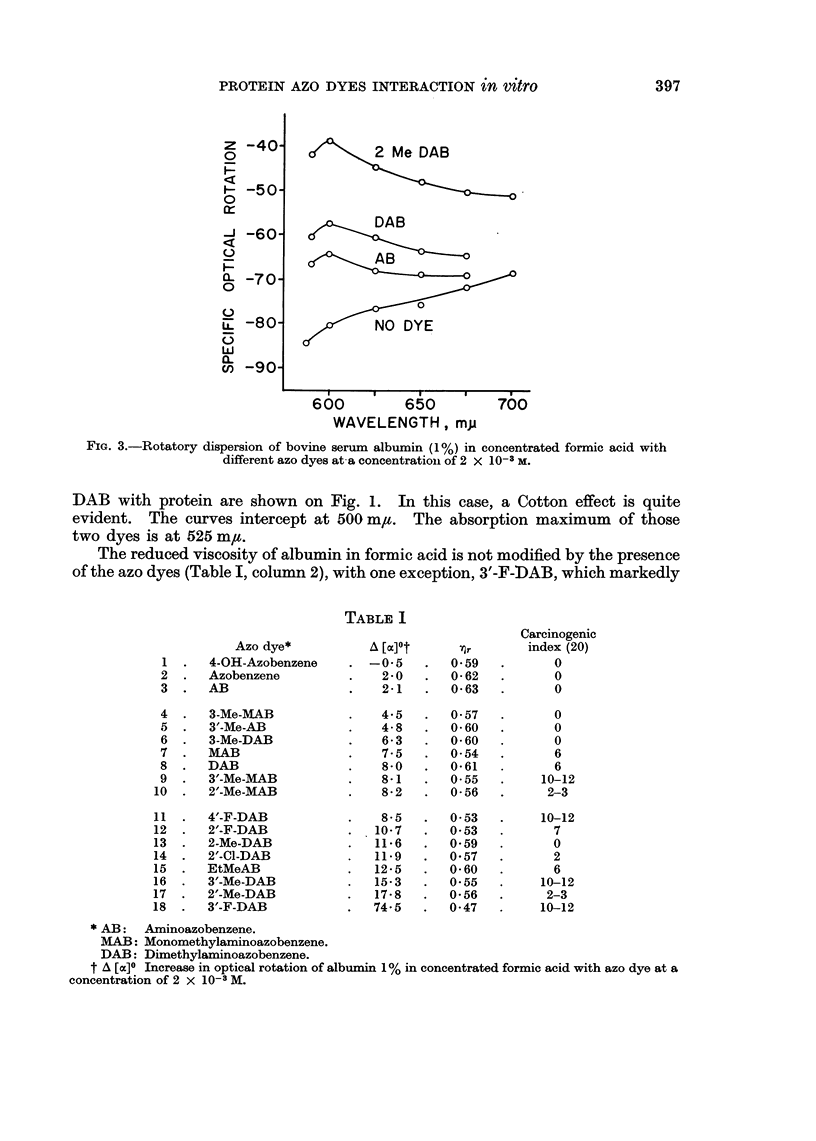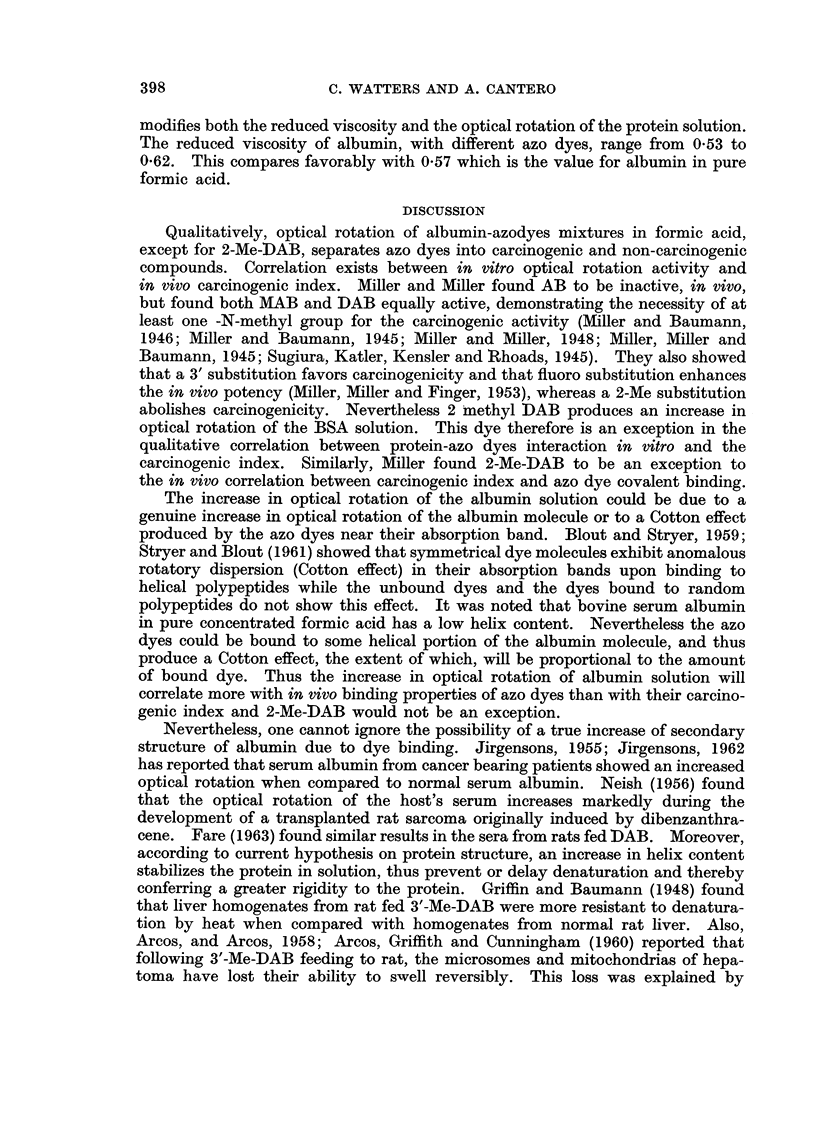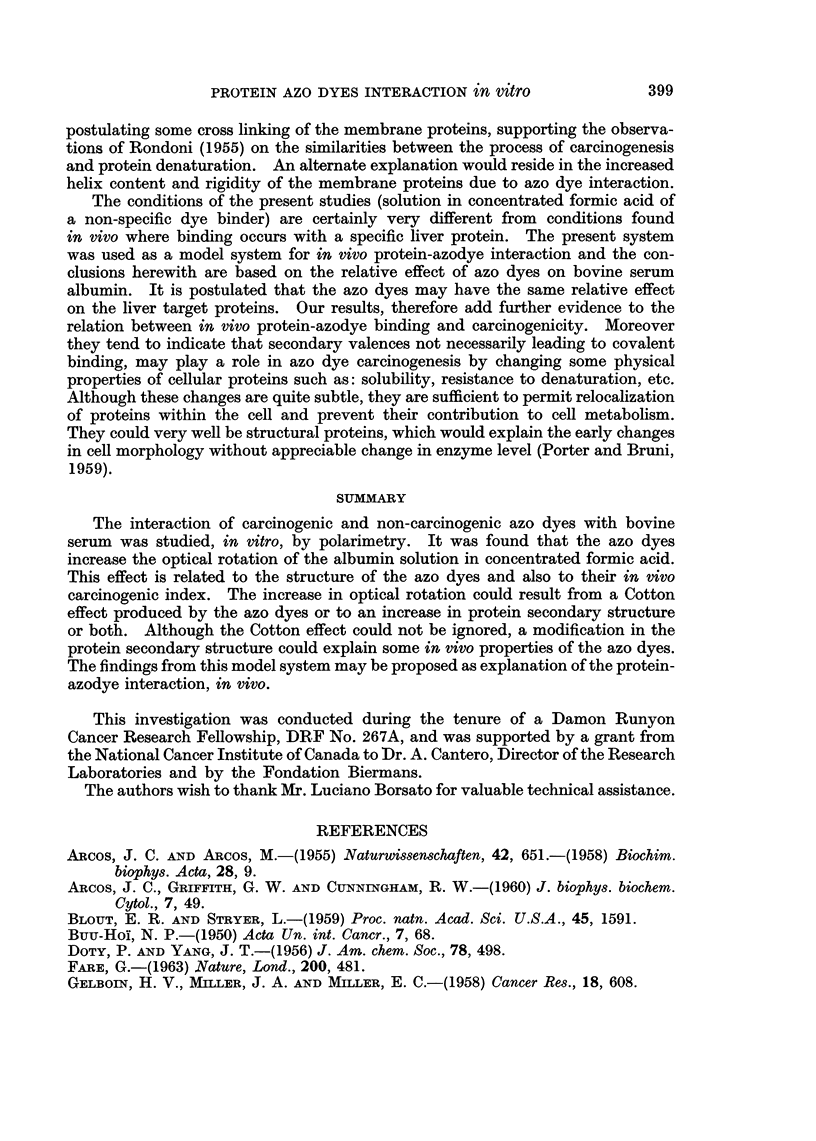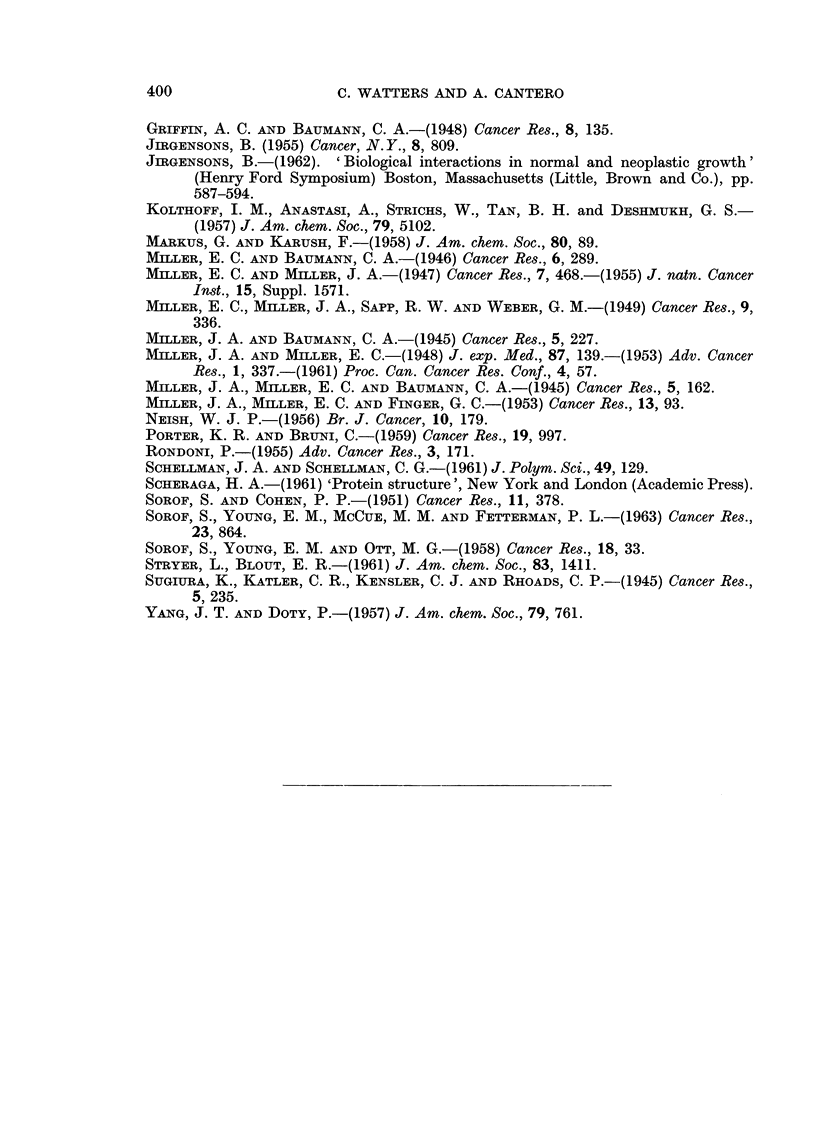# Protein Azo Dyes Interaction in vitro: Possible Role of Secondary Valences on Chemical Carcinogenesis

**Published:** 1967-06

**Authors:** C. Watters, A. Cantero


### PROTEIN AZO DYES INTERACTION *IN VITRO*: POSSIBLE ROLE OF SECONDARY VALENCES ON CHEMICAL CARCINOGENESIS

C. WATTERS AND A. CANTERO

*From the Institut du Cancer de Montréal, Laboratoires de Recherche, Hôpital Notre-Dame et Université de Montréal, Montréal, Canada*

Received for publication December 6, 1966

DURING azo dye hepatocarcinogenesis in the rat, the carcinogen is bound quite early to cellular proteins (Miller and Miller, 1947; Miller, Miller, Sapp and Weber, 1949; Miller and Miller, 1961). This binding occurs during protein synthesis and not on preformed macromolecules (Gelboin, Miller and Miller, 1958). The target proteins designated by Sorof as h-proteins (Sorof *et al.*, 1963) are basic in nature. They are found to be reduced in concentration in preneoplastic tissue and nearly absent from tumor (Sorof and Cohen, 1951; Sorof, Young, McCue and Fetterman, 1963; Sorof, Young and Ott, 1958) where no azo dye binding occurs (Miller and Miller, 1947). The nature and mechanism by which the h-proteins bind the azo dyes are still obscure. The deletion hypothesis (Miller and Miller, 1953) favors the view that the h-proteins are cellular in origin and are destroyed or inactivated by the azo dye binding, thus hindering or preventing the normal protein synthesis or inducing the synthesis of altered proteins through faulty duplication.

The nature of the protein-azo dye linkage is believed to be covalent (Miller and Miller, 1947). Some authors nevertheless have considered the participation of a weaker combination, by secondary valences, between protein and carcinogen, not necessarily involving firm binding (Miller and Miller, 1955). These forces range from van der Waals forces and hydrogen bonds to inclusion compounds (Arcos and Arcos, 1955; Buu-Hoï, 1950).

The present paper explores the effect of the weak protein-azodye interaction, *in vitro*, on spatial conformation of albumin, produced by carcinogenic and non-carcinogenic azo dyes.

Three different levels may be considered in the protein structure:

(a) The primary level involves the sequence of amino-acids in the polypeptide chain, the nature and the position of cross links which bridge different chains to form loops. This aspect is not the object of the present investigation.

(b) The second level considers the spatial conformation of the individual polypeptide chain or segments of chain which, according to current hypothesis, exists in solution in two basic conformations: as a regular cylindrical coil, probably the $\alpha$-helix; or, as an irregular structure, the random coil. The two types of secondary structure are in equilibrium and their proportion in a given protein may be modified by solvent, pH, denaturation, binding of small molecules, etc. and were found by Yang and Doty (Doty and Yang, 1956; Yang and Doty, 1957) to contribute independently to the optical rotation of the polypeptide. These investigators were therefore able to estimate the relative amounts of the helical structure in proteins by assuming that the random coil-helix transition increases the optical rotation of the macromolecule. In the present work, optical rotation was therefore chosen to follow the changes in protein secondary structure produced by the azo dyes.

(c) The third level considers the folds and turns which occur in the already coiled polypeptide chain in order to fit the entire molecule into a region of appropriate size and shape. Viscosity measurement is used in the present work to study this third level (tertiary structure), since any modification of the gross shape of a macromolecule modifies the macromolecular hydrodynamic properties (Scheraga, 1961).

### MATERIALS AND METHODS

Measurements reported in this paper were carried out with bovine serum albumin (BSA) and azo dyes dissolved in concentrated formic acid (90%). An original solution of BSA 1%, with an azo dye concentration of 2 mM, was prepared in formic acid. The optical rotation of this solution was measured, at room temperature, in a 4 dm tube, with a Bellingham and Stanley Polarimeter, which reads 0·01°. The standard error on the specific optical rotation is 2°. The concentration dependence of the azodye-protein interaction was studied by reading the optical rotation after stepwise dilutions of the original solution, with BSA 1% in formic acid.

To eliminate the Cotton effect at the absorption maximum of the dyes, measurements were performed at a wavelength outside the absorption band of the dye. This was achieved by using, as a light source, a tungsten filament lamp filtered through 5 cm. of a 2 mM solution of DAB in formic acid. This filter does not transmit below 600 m$\mu$ and shows 100% transmission at 650 m$\mu$. Rotary dispersion in the visible range was measured by using the above described polarimeter equipped with a Beckman UV monochromator as a light source.

Viscosity of the solution was measured by means of a suspended level Ubbelohde viscosimeter. The results of the viscosity experiments are expressed as reduced viscosity according to the equation:

$$\eta_r = 1/c\left(\frac{\eta}{\eta_0} - 1\right)$$

where $c$ is the concentration of albumin in %, $\eta$ is the viscosity of the solution and $\eta_0$ is the viscosity of the solvent.

Detection of reduced disulfide bonds in the albumin molecule was noted by measuring SH groups, by amperometric titration with formic acid as solvent, at the rotating platinum electrode (Kolthoff *et al.*, 1957).

The bovine serum albumin (cryst.) was purchased from Bios Laboratories, Inc., New York. The azo dyes were kindly supplied by Dr. J. A. Miller of the McArdle Memorial Laboratory for Cancer Research, Medical School, University of Wisconsin, Madison, Wisc.

### RESULTS

Bovine serum albumin in pure concentrated formic acid shows a sulfhydryl content of 0·66 ± 0·05 mole per mole of protein (molecular weight of 69,000). This is the same as for native BSA in aqueous solutions (Kolthoff *et al.*, 1957) indicating that formic acid preserves the integrity of the disulfide bonds of the protein. The specific optical rotation of BSA in concentrated formic acid is

$-75°$ at 620 m$\mu$. The dispersion curve, in the visible range (Fig. 1) follows a simple one term Drude equation

$$[\alpha]\lambda = \frac{K}{\lambda^2 - \lambda_c^2}$$

The value of $\lambda_c$ for BSA in concentrated formic acid is 208 m$\mu$. This value of $\lambda_c$, combined with dispersion obeying a one term Drude equation indicates that BSA in formic acid is a random coil or has a low helix content (Schellman and

FIG. 1.—Rotatory dispersion of bovine serum albumine (1%) in concentrated formic acid with different azo dyes at a concentration of $10^{-6}$ M.

Schellman, 1961). On the other hand, the reduced viscosity of BSA in formic acid is 0·57. This reveals that there occurs an expansion of the protein tertiary structure in formic acid since in aqueous solutions, BSA has a reduced viscosity of 0·04 (Kolthoff *et al.*, 1957).

The specificity optical rotation of a 1% solution of BSA in formic acid is altered by adding different azo dyes. This effect is concentration dependent as shown on Fig. 2 for nine of the eighteen azo dyes studied. The azo dyes produce an increase in the optical rotation of the albumin solution. Fig. 2A illustrates the effect of N-methylation of aminoazobenzene (AB). This azo dye (AB) has negligible effect on rotatory properties of albumin while the monomethyl (MAB) and dimethyl (DAB) derivatives are much more active, indicating that at least one N-methyl group is required for the azo dye to modify the optical rotation of albumin solution. At the highest concentration used (2 mM) MAB and DAB are equally active. Fig. 2B illustrates the effect of a 3′ Me substitution on AB, MAB and DAB. This ring substituent doubles the activity of the parent compounds. Fig. 2C shows the effect of a fluoro substitution on DAB. The 2′ and 4′ derivatives are as active as DAB but 3′–F–DAB is 7 to 8 times as active as DAB. Substitution therefore of an electron donating group (F or $CH_3$), especially in a 3′ position enhances the protein-azodye interaction. Table I compares all the azo dyes studied, at a concentration of 2 mM. The azo dyes are listed in order of their increasing effect on albumin optical rotation (column 1). With one exception (EtMeDAB), the 18 azo dyes may be separated into three groups. Azo dyes 11 to 18 inclusive have both a N-dimethyl group and an electron-donating substituent on the prime ring and thus having highest activity on the optical

FIG. 2.—Effect of different concentrations of azo dyes on specific optical rotation of bovine serum albumin (1%) in concentrated formic acid. The increase in specific optical rotation is expressed in degrees.

rotation of albumin. Azo dyes 4 to 10 inclusive lack one of these conditions and have an intermediate activity. All the N-monomethylated azo dyes belong to this group. Azo dyes 1, 2, and 3 form the third group, which lack the two conditions, and have negligible effect. The 4-hydroxyazobenzene is the only dye which decreases the optical rotation of albumin. Markus and Karush (1958) found a similar situation while studying the structural effects of anionic azo dyes on serum albumin. Their data showed that the hydroxyl group, in almost all cases, lowers the optical rotation of albumin, whereas the dimethylamino substituent increases the optical rotation.

The strong light absorption of the dyes unfortunately precluded the measurements of rotatory dispersion below a wavelength of 590 m$\mu$ with a 2 mM dye concentration. However measurements were carried out with 4 azo dyes (Fig. 3). Contrary to BSA in formic acid, the rotatory dispersion of the protein-azodye mixtures is strongly anomalous.

If the dye concentration is decreased to $10^{-6}$ M, the rotatory dispersion measurements could be extended to 450 m$\mu$. Such measurements for DAB and 2-Me-

FIG. 3.—Rotatory dispersion of bovine serum albumin (1%) in concentrated formic acid with different azo dyes at a concentration of $2 \times 10^{-3}$ M.

DAB with protein are shown on Fig. 1. In this case, a Cotton effect is quite evident. The curves intercept at 500 mμ. The absorption maximum of those two dyes is at 525 mμ.

The reduced viscosity of albumin in formic acid is not modified by the presence of the azo dyes (Table I, column 2), with one exception, 3′-F-DAB, which markedly

TABLE I

| | Azo dye* | $\Delta [\alpha]^0$† | $\eta_r$ | Carcinogenic index (20) |
|---|---|---|---|---|
| 1 | 4-OH-Azobenzene | −0·5 | 0·59 | 0 |
| 2 | Azobenzene | 2·0 | 0·62 | 0 |
| 3 | AB | 2·1 | 0·63 | 0 |
| 4 | 3-Me-MAB | 4·5 | 0·57 | 0 |
| 5 | 3′-Me-AB | 4·8 | 0·60 | 0 |
| 6 | 3-Me-DAB | 6·3 | 0·60 | 0 |
| 7 | MAB | 7·5 | 0·54 | 6 |
| 8 | DAB | 8·0 | 0·61 | 6 |
| 9 | 3′-Me-MAB | 8·1 | 0·55 | 10–12 |
| 10 | 2′-Me-MAB | 8·2 | 0·56 | 2–3 |
| 11 | 4′-F-DAB | 8·5 | 0·53 | 10–12 |
| 12 | 2′-F-DAB | 10·7 | 0·53 | 7 |
| 13 | 2-Me-DAB | 11·6 | 0·59 | 0 |
| 14 | 2′-Cl-DAB | 11·9 | 0·57 | 2 |
| 15 | EtMeAB | 12·5 | 0·60 | 6 |
| 16 | 3′-Me-DAB | 15·3 | 0·55 | 10–12 |
| 17 | 2′-Me-DAB | 17·8 | 0·56 | 2–3 |
| 18 | 3′-F-DAB | 74·5 | 0·47 | 10–12 |

* AB: Aminoazobenzene.
MAB: Monomethylaminoazobenzene.
DAB: Dimethylaminoazobenzene.
† $\Delta [\alpha]^0$ Increase in optical rotation of albumin 1% in concentrated formic acid with azo dye at a concentration of $2 \times 10^{-3}$ M.

modifies both the reduced viscosity and the optical rotation of the protein solution. The reduced viscosity of albumin, with different azo dyes, range from 0·53 to 0·62. This compares favorably with 0·57 which is the value for albumin in pure formic acid.

### DISCUSSION

Qualitatively, optical rotation of albumin-azodyes mixtures in formic acid, except for 2-Me-DAB, separates azo dyes into carcinogenic and non-carcinogenic compounds. Correlation exists between *in vitro* optical rotation activity and *in vivo* carcinogenic index. Miller and Miller found AB to be inactive, *in vivo*, but found both MAB and DAB equally active, demonstrating the necessity of at least one -N-methyl group for the carcinogenic activity (Miller and Baumann, 1946; Miller and Baumann, 1945; Miller and Miller, 1948; Miller, Miller and Baumann, 1945; Sugiura, Katler, Kensler and Rhoads, 1945). They also showed that a 3′ substitution favors carcinogenicity and that fluoro substitution enhances the *in vivo* potency (Miller, Miller and Finger, 1953), whereas a 2-Me substitution abolishes carcinogenicity. Nevertheless 2 methyl DAB produces an increase in optical rotation of the BSA solution. This dye therefore is an exception in the qualitative correlation between protein-azo dyes interaction *in vitro* and the carcinogenic index. Similarly, Miller found 2-Me-DAB to be an exception to the *in vivo* correlation between carcinogenic index and azo dye covalent binding.

The increase in optical rotation of the albumin solution could be due to a genuine increase in optical rotation of the albumin molecule or to a Cotton effect produced by the azo dyes near their absorption band. Blout and Stryer, 1959; Stryer and Blout (1961) showed that symmetrical dye molecules exhibit anomalous rotatory dispersion (Cotton effect) in their absorption bands upon binding to helical polypeptides while the unbound dyes and the dyes bound to random polypeptides do not show this effect. It was noted that bovine serum albumin in pure concentrated formic acid has a low helix content. Nevertheless the azo dyes could be bound to some helical portion of the albumin molecule, and thus produce a Cotton effect, the extent of which, will be proportional to the amount of bound dye. Thus the increase in optical rotation of albumin solution will correlate more with *in vivo* binding properties of azo dyes than with their carcinogenic index and 2-Me-DAB would not be an exception.

Nevertheless, one cannot ignore the possibility of a true increase of secondary structure of albumin due to dye binding. Jirgensons, 1955; Jirgensons, 1962 has reported that serum albumin from cancer bearing patients showed an increased optical rotation when compared to normal serum albumin. Neish (1956) found that the optical rotation of the host's serum increases markedly during the development of a transplanted rat sarcoma originally induced by dibenzanthracene. Fare (1963) found similar results in the sera from rats fed DAB. Moreover, according to current hypothesis on protein structure, an increase in helix content stabilizes the protein in solution, thus prevent or delay denaturation and thereby conferring a greater rigidity to the protein. Griffin and Baumann (1948) found that liver homogenates from rat fed 3′-Me-DAB were more resistant to denaturation by heat when compared with homogenates from normal rat liver. Also, Arcos, and Arcos, 1958; Arcos, Griffith and Cunningham (1960) reported that following 3′-Me-DAB feeding to rat, the microsomes and mitochondrias of hepatoma have lost their ability to swell reversibly. This loss was explained by postulating some cross linking of the membrane proteins, supporting the observations of Rondoni (1955) on the similarities between the process of carcinogenesis and protein denaturation. An alternate explanation would reside in the increased helix content and rigidity of the membrane proteins due to azo dye interaction.

The conditions of the present studies (solution in concentrated formic acid of a non-specific dye binder) are certainly very different from conditions found *in vivo* where binding occurs with a specific liver protein. The present system was used as a model system for *in vivo* protein-azodye interaction and the conclusions herewith are based on the relative effect of azo dyes on bovine serum albumin. It is postulated that the azo dyes may have the same relative effect on the liver target proteins. Our results, therefore add further evidence to the relation between *in vivo* protein-azodye binding and carcinogenicity. Moreover they tend to indicate that secondary valences not necessarily leading to covalent binding, may play a role in azo dye carcinogenesis by changing some physical properties of cellular proteins such as: solubility, resistance to denaturation, etc. Although these changes are quite subtle, they are sufficient to permit relocalization of proteins within the cell and prevent their contribution to cell metabolism. They could very well be structural proteins, which would explain the early changes in cell morphology without appreciable change in enzyme level (Porter and Bruni, 1959).

### SUMMARY

The interaction of carcinogenic and non-carcinogenic azo dyes with bovine serum was studied, *in vitro*, by polarimetry. It was found that the azo dyes increase the optical rotation of the albumin solution in concentrated formic acid. This effect is related to the structure of the azo dyes and also to their *in vivo* carcinogenic index. The increase in optical rotation could result from a Cotton effect produced by the azo dyes or to an increase in protein secondary structure or both. Although the Cotton effect could not be ignored, a modification in the protein secondary structure could explain some *in vivo* properties of the azo dyes. The findings from this model system may be proposed as explanation of the protein-azodye interaction, *in vivo*.

This investigation was conducted during the tenure of a Damon Runyon Cancer Research Fellowship, DRF No. 267A, and was supported by a grant from the National Cancer Institute of Canada to Dr. A. Cantero, Director of the Research Laboratories and by the Fondation Biermans.

The authors wish to thank Mr. Luciano Borsato for valuable technical assistance.

### REFERENCES

ARCOS, J. C. AND ARCOS, M.—(1955) *Naturwissenschaften*, **42**, 651.—(1958) *Biochim. biophys. Acta*, **28**, 9.
ARCOS, J. C., GRIFFITH, G. W. AND CUNNINGHAM, R. W.—(1960) *J. biophys. biochem. Cytol.*, **7**, 49.
BLOUT, E. R. AND STRYER, L.—(1959) *Proc. natn. Acad. Sci. U.S.A.*, **45**, 1591.
BUU-HOÏ, N. P.—(1950) *Acta Un. int. Cancr.*, **7**, 68.
DOTY, P. AND YANG, J. T.—(1956) *J. Am. chem. Soc.*, **78**, 498.
FARE, G.—(1963) *Nature, Lond.*, **200**, 481.
GELBOIN, H. V., MILLER, J. A. AND MILLER, E. C.—(1958) *Cancer Res.*, **18**, 608.

GRIFFIN, A. C. AND BAUMANN, C. A.—(1948) *Cancer Res.*, **8**, 135.
JIRGENSONS, B. (1955) *Cancer, N.Y.*, **8**, 809.
JIRGENSONS, B.—(1962). 'Biological interactions in normal and neoplastic growth' (Henry Ford Symposium) Boston, Massachusetts (Little, Brown and Co.), pp. 587–594.
KOLTHOFF, I. M., ANASTASI, A., STRICHS, W., TAN, B. H. and DESHMUKH, G. S.—(1957) *J. Am. chem. Soc.*, **79**, 5102.
MARKUS, G. AND KARUSH, F.—(1958) *J. Am. chem. Soc.*, **80**, 89.
MILLER, E. C. AND BAUMANN, C. A.—(1946) *Cancer Res.*, **6**, 289.
MILLER, E. C. AND MILLER, J. A.—(1947) *Cancer Res.*, **7**, 468.—(1955) *J. natn. Cancer Inst.*, **15**, Suppl. 1571.
MILLER, E. C., MILLER, J. A., SAPP, R. W. AND WEBER, G. M.—(1949) *Cancer Res.*, **9**, 336.
MILLER, J. A. AND BAUMANN, C. A.—(1945) *Cancer Res.*, **5**, 227.
MILLER, J. A. AND MILLER, E. C.—(1948) *J. exp. Med.*, **87**, 139.—(1953) *Adv. Cancer Res.*, **1**, 337.—(1961) *Proc. Can. Cancer Res. Conf.*, **4**, 57.
MILLER, J. A., MILLER, E. C. AND BAUMANN, C. A.—(1945) *Cancer Res.*, **5**, 162.
MILLER, J. A., MILLER, E. C. AND FINGER, G. C.—(1953) *Cancer Res.*, **13**, 93.
NEISH, W. J. P.—(1956) *Br. J. Cancer*, **10**, 179.
PORTER, K. R. AND BRUNI, C.—(1959) *Cancer Res.*, **19**, 997.
RONDONI, P.—(1955) *Adv. Cancer Res.*, **3**, 171.
SCHELLMAN, J. A. AND SCHELLMAN, C. G.—(1961) *J. Polym. Sci.*, **49**, 129.
SCHERAGA, H. A.—(1961) 'Protein structure', New York and London (Academic Press).
SOROF, S. AND COHEN, P. P.—(1951) *Cancer Res.*, **11**, 378.
SOROF, S., YOUNG, E. M., MCCUE, M. M. AND FETTERMAN, P. L.—(1963) *Cancer Res.*, **23**, 864.
SOROF, S., YOUNG, E. M. AND OTT, M. G.—(1958) *Cancer Res.*, **18**, 33.
STRYER, L., BLOUT, E. R.—(1961) *J. Am. chem. Soc.*, **83**, 1411.
SUGIURA, K., KATLER, C. R., KENSLER, C. J. AND RHOADS, C. P.—(1945) *Cancer Res.*, **5**, 235.
YANG, J. T. AND DOTY, P.—(1957) *J. Am. chem. Soc.*, **79**, 761.